# Infectious clones of *Tomato leaf curl Palampur virus *with a defective DNA B and their pseudo-recombination with *Tomato leaf curl New Delhi virus*

**DOI:** 10.1186/1743-422X-8-173

**Published:** 2011-04-15

**Authors:** Aamir Humayun Malik, Rob W Briddon, Shahid Mansoor

**Affiliations:** 1 Agricultural Biotechnology Division, National Institute for Biotechnology and Genetic Engineering, P O Box 577, Jhang Road, Faisalabad, Pakistan

## Abstract

**Background:**

*Tomato leaf curl Palampur virus *(ToLCPMV) is a bipartite begomovirus which has been reported from India and Iran but infectious clones have not been obtained. We have previously shown the association of *Zucchini yellow mosaic virus *(ZYMV), a potyvirus, with severe leaf curl disease of muskmelon in Pakistan. However, the severity of symptoms in the field and yield losses led us to believe that some other agent, such as a begomovirus, could be associated with the disease.

**Results:**

A bipartite begomovirus associated with a severe yellow leaf curl disease on muskmelon in Pakistan has been characterized. Analysis of the complete nucleotide sequence of the DNA A and DNA B components of the begomovirus showed that it has the highest DNA sequence identity with ToLCPMV. However, the gene encoding the nuclear shuttle protein (NSP) was truncated in comparison to previously characterised isolates. *Agrobacterium*-mediated inoculation of *Nicotiana benthamiana *with the ToLCPMV clones obtained here did not result in symptoms. However, inoculation of plants with the DNA A component of ToLCPMV and the DNA B component of *Tomato leaf curl New Delhi virus *(ToLCNDV) lead to systemic infection with leaf curl symptoms. This suggested that the lack of infectivity of the ToLCPMV clones was due to the defect in DNA B. The DNA B of ToLCPMV was able to move systemically when inoculated with DNA A of the either virus. Agro-infiltration of muskmelon with the DNA A and DNA B components of ToLCPMV did not lead to symptomatic infection whereas inoculation with the DNA A with the DNA B of ToLCNDV resulted in a hypersensitive response (HR) along the veins. Additionally, agro-infiltration of muskmelon with a construct for the expression of the NSP gene of ToLCNDV under the control of the cauliflower mosaic virus 35S promoter induced a HR, suggesting that this is the gene causing the HR.

**Conclusions:**

Both ToLCPMV and ZYMV are associated with muskmelon leaf curl disease in Pakistan. However, the ToLCPMV variant identified in association with ZYMV has a defective NSP. The results suggest that a variant with a defective NSP may have been selected for in muskmelon, as this protein is an avirulence determinant in this species, and possibly that infection requires the synergistic interaction with ZYMV.

## Background

Whitefly-transmitted geminiviruses have emerged as major pathogens of food and fiber crops throughout the world, particularly in tropical and sub-tropical regions [1]. Viruses of the family *Geminiviridae *have circular single-stranded (ss) DNA genomes, encapsidated in characteristic twinned isometric particles. These viruses are classified into four genera, *Mastrevirus*, *Curtovirus*, *Topocuvirus *and *Begomoviru*s [2] depending upon their insect vector, host range and genome organization. Begomoviruses are exclusively transmitted by the whitefly *Bemisia tabaci *and infect only dicotyledonous plants. They may be monopartite or bipartite (having genomes consisting of one or two circular single-stranded DNA molecules) [3]. Since the identification of the betasatellites [4], it has become apparent that the majority of begomoviruses in the Old World are monopartite and associate with betasatellites, asymptom modulating ssDNA satellite [5-7].

Several factors, including multiple infections, recombination and interspecific synergism, appear to be the major cause of the increased importance of begomoviruses to agriculture in recent years. Intriguingly, begomovirus-betasatellite complexes do not appear to be a problem in cucurbits; only a single defective betasatellite isolated from a cucurbit has been reported so far [8]. The DNA A and DNA B components of begomoviruses encode genes both in the virion and complementary-sense orientations. Old World begomoviruses encode four open reading frames (ORFs) in the complementary-sense that are involved in viral DNA replication and control of gene expression, while two ORFs in the virion-sense are involved in virus encapsidation and movement. The two proteins encoded by the DNA B component are the nuclear shuttle protein (NSP) and the movement protein (MP) that are involved in nuclear transport and cell-to-cell movement of viral DNA, respectively [9].

Both watermelon and muskmelon form an important part of diet in Pakistan, particularly in early summer. These crops fit nicely in the cropping pattern where vegetables are grown in the winter months when land is available before the start of cotton growing season. However, the traditional muskmelon-growing areas have recently been hit by a severe viral disease. We have previously shown that a potyvirus, *Zucchini yellow mosaic virus *(ZYMV), is associated with the disease of muskmelon [10]. Here, we have characterized a bipartite begomovirus associated with severe leaf curl disease on muskmelon in Pakistan.

## Methods

### Collection of samples and DNA extraction

Samples were collected as described earlier [10]. Total DNA was isolated from leaf samples using CTAB method [11].

### Detection and cloning of virus components

PCR-mediated detection of begomoviruses in DNA samples extracted from symptomatic leaves was done by using two sets of universal primer pairs (Begomo1/Begomo2 and Begomo3/Begomo4; Table 1) designed for amplification of different length fragments [12]. Full-length length PCR product of DNA A was amplified by using primers Melonfor and Melonrev (Table: 1). Oligonucleotide primers used for amplification of complete DNA B were TLCVB F and TLCVB R (Table: 1). PCR was performed under standard conditions as described previously [13]. Primer pairs used for amplification of MP, NSP and full-length DNA B are also shown in Table 1. Samples were also tested for the presence of DNA B of begomoviruses using a PCR product (produced using primer pair MPF/MPR; Table 1), encompassing the MP gene of *Tomato leaf curl New Delhi virus *(ToLCNDV-IN[IN:ND:Svr:92])(acc. no U15017), as a probe for ToLCNDV DNA B [14]. The probe was prepared using a Hexalabeling DNA labeling kit (Fermentas) and [α-^32^P]dCTP (Amersham, UK). Hybridization was carried out overnight at 65°C in a hybridization oven. Post-hybridization washes were conducted at medium-stringency.

**Table 1:** Primers used to amplify partial and full length genome sequences of begomovirus components.

Primer	Sequence	Expected fragment size (kb)	Target Component/gene
Begomo1	CCGTGCTGCTGCCCCCATTGTCCGCGTCAC	1.1	DNA A
Begomo2	CTGCCACAACCTGGATTCACGCACAGGG	1.1	DNA A
Begomo3	GTTCCCTGTGCGTGAATCCATGGTTGTGG	1.7	DNA A
Begomo4	TTTTGTGACGCGGACA ATGGGGGCAGCA	1.7	DNA A
Melonfor	GGTACCTAAGGAC CTGGGTTCTG	2.8	DNA A
Melonrev	GGTACCTGGATATGCTAGGTGTTATAG	2.8	DNA A
TLCVB F	GCTAAGCT TCTGCTCGAACATGGATGGAA	2.7	DNA B
TLCVB R	CAGAAGCTTAGCCAGTTGAGGAATAGATG	2.7	DNA B
MLU F	ATACGCGTAAGGAAATCTGTGAAACAC	2.7	DNA B
MLU R	CCTTACGCGTATATTGTTTGGAGATT	2.7	DNA B
Pst F	GCTCTGCAGCATCGTTTGTGAGAGCG	2.7	DNA B
Pst R	CGTCTGCAGAAGTTACCTTGTCATTTCC	2.7	DNA B
MP F	CACCATGGCAATAGGAAATGATGGTATGGG		MP
MP R	AAGGATCCTCTTATTTTTTGAATAAATTTGG		MP
NSP F	GGCATCGATGGTGTTTCCGTTGCCACAC		NSP
NSP R	GGCGTCGACATATCCAATGTAATTAAGAATT		NSP

### Sequencing and sequence analysis

The complete nucleotide sequences of clones were determined by dideoxynucleotide chain-termination sequencing using the PCR-based BIG DYE kit (Perkin-Elmer Cetus) and specific internal primers. Sequencing products were resolved commercially (Macrogen, Korea). Sequence information was stored, assembled and analysed using the Lasergene sequence analysis package (DNAStar Inc., Madison, WI, USA) running on an IBM-compatible PC. Phylogenetic analyses were conducted on matrices of aligned sequences using the neighbour-joining and bootstrap options of Phylip (ver. 3.5c) running on an IBM-compatible personal computer. Sequence alignments were produced using CLUSTAL X [15]. Phylogenetic dendrograms were viewed, manipulated and printed using Treeview [16].

### Agroinoculation of cloned DNA

Partial direct repeat constructs for the *Agrobacterium*-mediated inoculation of the full-length DNA A and DNA B clones were produced in the binary vector pGreen0029 [17]. An approximately 1680 bp fragment of the DNA A and an approximately 823 bp fragment of the DNA B clones were released from the full-length pTZ57/RT clones by digestion with *Kpn*I and *Bam*HI or *Hind*III and *Mlu*I, respectively, and ligated into suitably digested pGreen0029 vector. The resulting pGreen0029 constructs were then linearised using *Kpn*I and *Hind*III, respectively, and the full-length fragments (release from the pTZ57/RT clones by digestion with *Kpn*I and *Hind*III, respectively) ligated into these to yield the partial, direct repeat constructs. The production of constructs in the binary vector pBin19 for inoculation of ToLCNDV has been described previously [18]. These binary vector constructs were transformed into *Agrobacterium tumefaciens *GV1301 and inoculated to plants by infiltration as described previously [18].

## Results

### A bipartite begomovirus is associated with severe disease on melon

As described earlier, a very severe yellow leaf curl disease was observed in several districts of central and southern Punjab, including Vehari, Sahiwal and Khanewal, and was shown to be associated with *Zucchini yellow mosaic virus *(ZYMV) [10]. Total DNA isolated from symptomatic plants was used as template in PCR with universal primers designed for the amplification of the partial sequences of begomovirus DNA A. The amplified product was cloned and sequenced and it showed the highest levels of nucleotide sequence identity to isolates of *Tomato leaf curl Palampur virus *(ToLCPMV), a bipartite begomovirus. To confirm the bipartite nature of virus from muskmelon, a PCR product encompassing the movement protein (MP) gene of ToLCNDV was used as a specific probe for Southern hybridisation. All the muskmelon samples found positive for DNA A were also found positive for DNA B (results not shown). Samples were similarly examined for the presence of begomoviruses by Southern blot hybridization using the DNA A of ToLCNDV as a general probe for begomoviruses. The probe hybridized with all sixty samples collected from symptomatic plants that were found positive by PCR.

### Cloning and complete nucleotide sequence of DNA A

**Figure 1:**
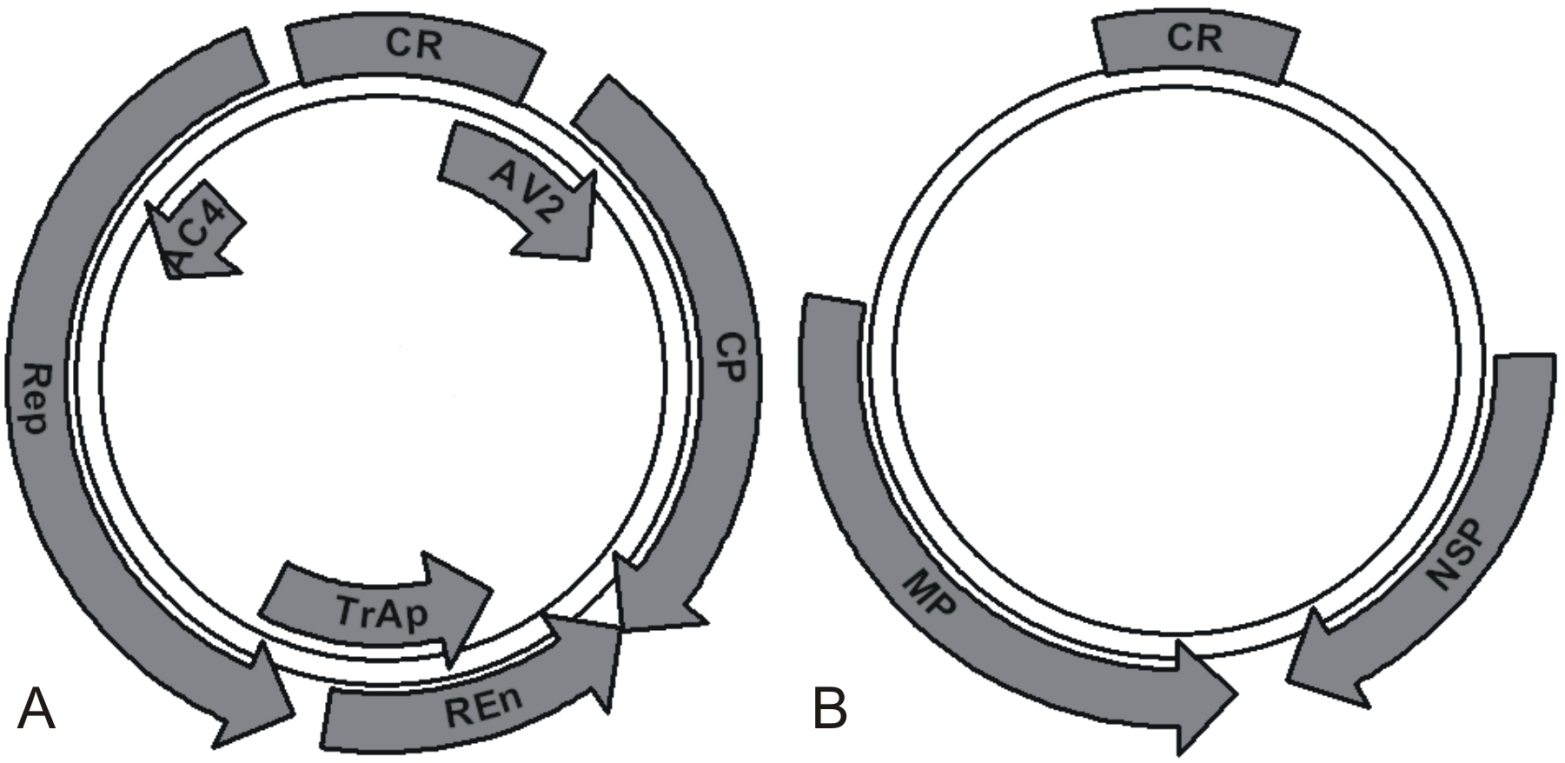
**Genome organization of ToLCPMV DNA A and DNA B**. The positions and orientations of predicted ORFs are shown with arrows. The common region (CR) of each component of the virus is indicated by a grey box. The position of the stem-loop structure, containing the conserved nonanucleotide loop sequence (TAATATTAC), is indicated by a black circle.

**Figure 2:**
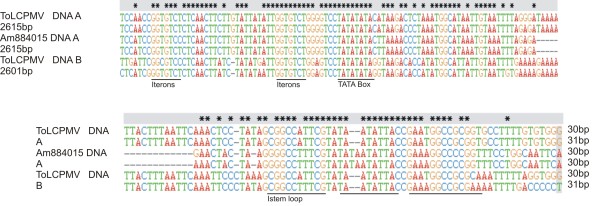
**Alignment of the intergenic region sequences of DNA A and DNA B components of ToLCPMV and ToLCNDV**. Conserved sequences in the alignment are marked (*). Gaps (-) were introduced into the sequences to optimize the alignment. The positions of the stem-loop, conserved nonanucleotide sequences, the TATA box of the Rep promoter and predicted iterons are indicated underlined.

Based upon the partial sequences obtained, back-to-back primers for amplification of full-length DNA A (MelF and MelR; Table: 1) were designed to the coat protein region on a naturally present, unique *Kpn*I restriction site. A full-length product (approximately 2.7 kb) was obtained and cloned in pTZ57RT. Restriction of the clone with *Kpn*I resulted in a single band on the gel whereas restriction of clone with *Sma*I linearized the clone and a 5.6 kb band appeared on the gel. The potentially full-length clone was sequenced in both orientations. A sequence similarity search (Blast) was performed by comparing the sequence to other begomovirus sequences in the database. The results showed that the sequence has a high level of identity to begomoviruses from Asia. The DNA A comprised of 2756 bp and showed the highest levels of nucleotide sequence identity (97%) to a ToLCPMV isolate from India (accession no. AM884015) and 96% identity to an isolate from Iran (accession no. FJ660444). The sequence identity with ToLCPMV isolates ranged from 96-97% whereas to isolates of ToLCNDV the highest sequence identity (86.7%) was with an isolate from Rahim Yar Khan, Pakistan (accession no. DQ116885). On the basis of DNA A sequence identity, it is clear that the muskmelon infecting begomovirus in Pakistan is an isolate of ToLCPMV, a begomovirus species previously reported infecting tomato in India and Iran [19,20]. Sequence analysis showed that it is a typical Old World begomovirus and encodes four open reading frames (Rep, REn, TrAP and AC4) in the complementary-sense and two (V2 and coat protein) in the virion-sense (Table 2), separated by an intergenic region (IR) (Figure 1A). The IR contains the conserved nonanucleotide motif (TAATATTAC), forming part of the loop of a predicted stem-loop structure found in all geminiviruses, a TATA motif (a sequence found in the promoter region and binding site for transcription factors) [21] (2664-2667 nt) and iteron sequences (GGTGTC) 5' of the TATA motif (Figure 2) [21,22]. The proteins predicted to be encoded by the DNA A component were of the sizes expected for begomovirus DNA A encoded proteins (Table: 2) [14].

**Table 2:** Positions and coding capacities of genes encoded by ToLCPMV DNA A and DNA B

Component	Gene	Position of start codon	Position of stop codon	Size (number of nucleotides)	Predicted size of encoded product (kDa)
DNA A	AV2	120	458	338	13.2
	CP	280	1050	771	29.8
	REn	1457	1047	411	16.0
	TrAp	1596	1177	410	15.9
	Rep	2602	1499	1104	41.6
	AC4	2445	2269	177	6.7

DNA B	NSP	681	1235	555	20.9
	MP	2146	1301	884	32.0

### Cloning and sequence analysis of DNA B

The partial sequences of DNA B obtained were used to design back-to-back primers (TLCVBF and TLCVBR; Table: 1) for the amplification of the full-length DNA B within the sequence of the movement protein (MP) gene. The desired sized fragment (2.7 kb) of DNA B was cloned in pTZ57RT. The clone was fully sequenced and shown to consist of 2724 bp. The DNA B showed 98% nucleotide sequence identity to an isolate of ToLCPMV recorded from India (accession no. AM992534) whereas the identity to the ToLCPMV isolate from Iran was 90% (accession no. FJ660442). Among the DNA B components of ToLCNDV available in the databases the highest levels of identity (82%) were to an isolate from India obtained from pumpkin (accession no. AM286435). In ORFs, NSP showed 99% sequence identity with ToLCPMV (accession no. AM992534) and it ranged from 90-99%. Whereas it was 73.3% identical to ToLCNDV (accession no AY150305). The maximum sequence identity to ToLCPMV isolate originating from India was 99% but to the isolates originating from Iran it was 95%. Overall among ToLCPMV isolates, sequence identity ranged from 92-99%. ToLCPMV MP was 86% identical to MP of ToLCNDV (accession no AY150304). Thus, DNA B might be a recombinant molecule where the MP is derived from DNA B of ToLCNDV while the intergenic region and NSP are derived from yet an unknown virus. The intergenic region of ToLCPMV shows 86% nucleotide sequence identity to Indian isolate (accession no. AM992534) whereas the identity to Iranian isolate is 76% (accession no. FJ660442). NSP has a size of 555 nucleotides (184 amino acids) and it is truncated at the N-terminus, since first 84 amino acids are missing in comparison to the ToLCNDV sequences available in the database (Figure 1B). The MP has a predicted size of 884 bases (294 amino acids) (Table: 2). Non-coding IR sequences of DNA B are distinct from already published sequences. The intergenic regions of DNA A and DNA B show only 38.2% identity but share a 34-bp potential stem-loop forming region (GGCCATTCGTATAATATTACCGAATGGCCGCGGT). This sequence has the conserved nonanucleotide sequence (TAATATTAC). The iterated elements (iterons) were close to a TATA box in the common region and were identified as GGTGTC (Figure 2) being the same as those identified in DNA A of ToLCPMV and ToLCNDV [22].

Efforts were made to identify a DNA B in muskmelon having no truncation of the NSP gene. Three set of primers were designed in the NSP and non-coding sequences of DNA B of ToLCNDV (Table: 1). None of the primer sets was able to amplify a product of approximately 2.8 kb. The ability of these sets of primers to amplify full-length DNA B was confirmed by the use of these primers on tomato samples infected experimentally with ToLCNDV. All sets of primers amplified products of the expected size. To further rule out the possible presence of other DNA B, phi29 polymerase was used which has the ability to amplify circular molecules. The amplified product was restricted with *Pst*I endonuclease (for which there are two restriction sites in DNA A) yielding two bands of the expected sizes (969 bases and 1786 bases). The DNA B of ToLCPMV has two *Pst*I restriction sites that are separated by 62 bases. The restriction analysis suggested the presence of a single type of DNA B of ToLCPMV and the absence of DNA B of ToLCNDV (results not shown).

### Phylogenetic analysis of ToLCPMV DNA A and DNA B

**Figure 3:**
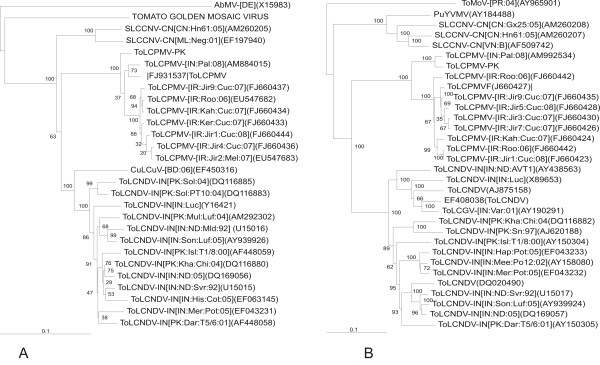
**Neighbour joining phylogenetic dendrogram based upon an alignment of ToLCPMV DNA components**. The viruses used in phylogenetic analysis are Abutilon mosaic virus (AbMV), Tomato mottle virus (ToMoV), Tomato leaf curl New Delhi virus (ToLCNDV), Squash leaf curl China virus (SLCCNV), Tomato leaf curl Gujrat virus (ToLCGV), Cucumber leaf curl virus (CuLCuV), Luffa yellow mosaic virus (LYMV), Squash leaf curl Philippines virus (SLCuPV). The phylogenetic trees are based on complete nucleotide sequences of ToLCPMV DNA A (Fig. 3A), complete nucleotide sequences of ToLCPMV DNA B (Fig. 3B). Numbers at the nodes are percentage bootstrap confidence values (1000 replicates). Vertical lines are arbitrary and horizontal lines are proportional to calculated mutation distances.

A phylogenetic analysis, based upon an alignment of the complete DNA A sequence of ToLCPMV with selected other begomoviruses is shown in Figure 3A. This analysis shows ToLCPMV to segregate with ToLCPMV isolates reported from India [19] and Iran [20] followed by *Cucumber leaf curl virus *(CuLCuV-[BD:06](acc. no. EF450316); a begomovirus recently identified in Bangladesh). These two viruses group with, and are basal to, the ToLCNDV isolates but distinct from *Squash leaf curl China virus *(SLCCNV) isolates. This is well supported by bootstrapping. A phylogenetic analysis based upon an alignment of the complete DNA B sequence of ToLCPMV with selected other begomoviruses is shown in Figure 3B. In this analysis ToLCPMV is again basal to the ToLCNDV isolates which form a clade distinct from the SLCCNV isolates. It is also noteworthy that the DNA B of *Tomato leaf curl Gujarat virus *(ToLCGV-[IN:Var:01]) is included in this analysis and shows it to have a ToLCNDV-like DNA B, as discussed later. The position of ToLCPMV in the tree is well supported by bootstrapping.

### Infectivity of ToLCPMV clones from muskmelon

**Figure 4:**
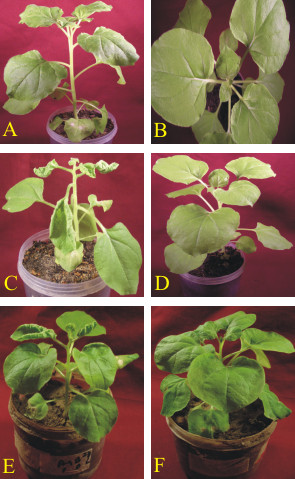
**Virus replication in systemic leaves of *N. benthamiana *inoculated with virus constructs in different combinations**. *N. benthamiana *plants agroinoculated with ToLCPMV DNA A (A), ToLCPMV DNA A and DNA B (B) and ToLCPMV DNA A and ToLCNDV DNA B (C). (D) plant only inoculated with *A. tumefaciens *without vector construct, ToLCNDV DNA A and DNA B (E), ToLCNDV DNA A and ToLCPMV DNA B (F). The plants were photographed 21 days post-inoculation.

The infectivity of ToLCPMV clones was assessed by *Agrobacterium*-mediated inoculation to *Nicotiana benthamiana *(Figure 4 and Table: 3). Plants inoculated with ToLCPMV DNA A alone (Figure 4A) or ToLCPMV DNA A and DNA B did not develop symptoms (Figure 4B), even 2 months post-inoculation. However, diagnostic PCR showed the presence of both ToLCPMV DNA A and DNA B in systemic leaves.. In contrast, plants inoculated with ToLCNDV DNA A and DNA B developed upward leaf curl symptoms and vein swelling within 14 days of inoculation (Figure 4E). These symptoms are typical of ToLCNDV in *N. benthamiana *[18]. The inability of ToLCPMV clones to cause disease symptoms in *N. benthamiana *indicates that they are competent for systemic infection but unable to produce disease symptoms.

ToLCPMV DNA A and DNA B were inoculated by biolistic methods. However, biolistic inoculation did not result in typical disease symptoms. PCR with specific primers was used to confirm systemic infection. The results showed the presence of both DNA A and DNA B in systemically infected leaves. This result shows that DNA B of ToLCPMV is capable of movement with ToLCPMV DNA A although the clone was not capable of inducing typical disease symptoms.

### ToLCNDV DNA B will support symptomatic infection of ToLCPMV DNA A

To ascertain which of the components was responsible for the lack of infectivity, the ToLCPMV clones were inoculated as pseudo-recombinants with the components of ToLCNDV. Inoculation of twenty *N. benthamiana *plants with ToLCNDV DNA A and ToLCPMV DNA B did not develop begomovirus symptoms (Figure 4F) but seven plants were found positive in PCR for the presence of DNA B. In contrast, all the plants inoculated with ToLCPMV DNA A and ToLCNDV DNA B developed symptoms typical of ToLCNDV including upward leaf curl and vein swelling symptoms 14 days post-inoculation (Table 3; Figure 4C). These results demonstrate that the defect(s) in symptom induction by ToLCPMV clones resides on DNA B. The earlier sequence analyses indicated that ToLCPMV DNA B contains a truncation of the NSP gene due to a premature stop codon. This is the likely cause of the lack of ability of ToLCPMV DNA B to support symptoms development. The ability of ToLCPMV to trans-replicate and to establish a systemic infection with ToLCNDV also indicates that the Rep encoded by ToLCPMV DNA A is able to interact with the origin of replication of ToLCNDV. The sequence analysis showed that the predicted iteron sequences of ToLCPMV are the same as iterons of ToLCNDV [14] and these elements are crucial in the interactions required for Rep to initiate rolling-circle replication [24].

**Table 3:** Infectivity of ToLCPMV and ToLCNDV clones to *N. benthamiana *

Inoculum	Infectivity (no. of plants showing symptoms/no. of plants inoculated)		
	
	Exp. I	Exp. II	Exp. III
ToLCPMV DNA A + ToLCPMV DNA B	0/20	0/20	0/20
ToLCPMV DNA A + ToLCNDV DNA B	20/20	20/20	20/20
ToLCNDV DNA A + ToLCPMV DNA B	0/20	0/20	0/20
ToLCNDV DNA A + ToLCNDV DNA B	20/20	20/20	20/20
ToLCPMV DNA A	0/20	0/20	0/20
ToLCNDV DNA A	0/20	0/20	0/20

### Inoculation of ToLCPMV DNA A with ToLCNDV DNA B induces a hypersensitive response in muskmelon

**Figure 5:**
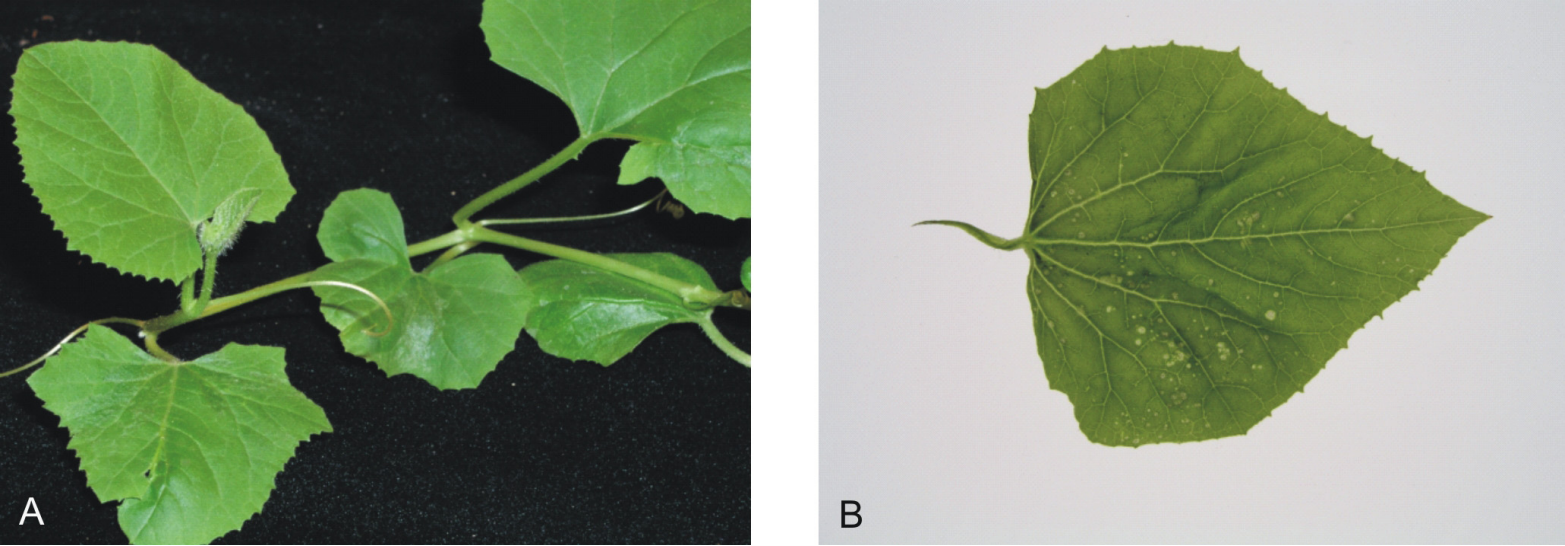
**Inoculation of muskmelon plant with ToLCPMV DNA A ToLCNDV DNA**. Cell death along the veins of systemic leaves of a muskmelon plant agroinoculated with ToLPMV DNA A and DNA B (A). Necrotic lesions along the veins of systemic leaf of a muskmelon plant micrografted with leaf discs of a *N. benthamiana *plant infected with ToLCPMV DNA A and ToLCNDV DNA B by agroinoculation (B).

**Figure 6:**
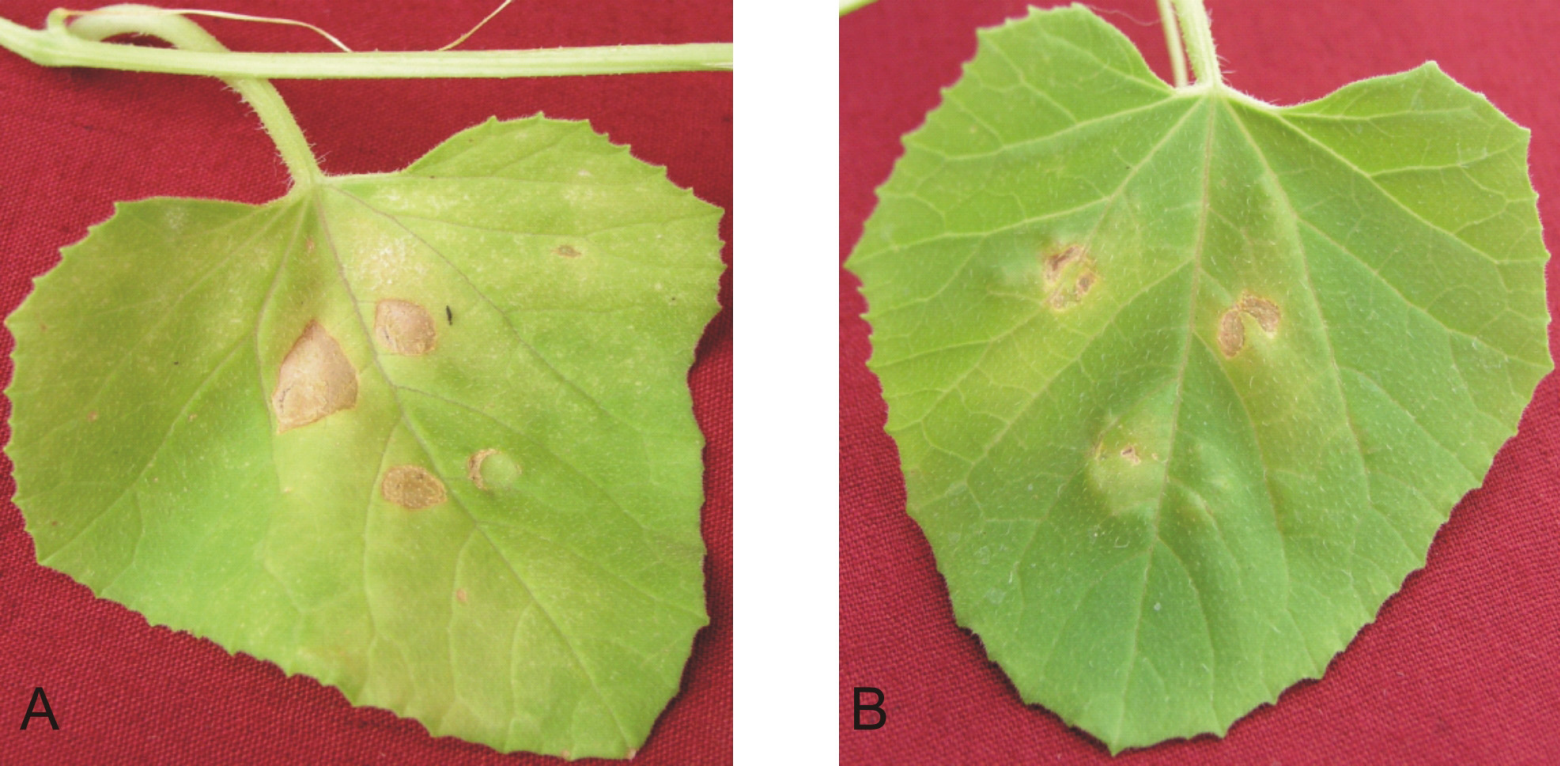
**Agroinfiltration of NSP and MP cloned under 35S promoter**. Hypersensitive cell death around the inoculation site of a muskmelon leaf agroinfiltrated with ToLCNDV NSP expressed under control of 35S promoter (A). Inoculation site of a muskmelon leaf agroinfiltrated with ToLCNDV MP expressed from a PVX vector (B). The necrosis in this case does not extend to all inoculated cells and is due to damage during infiltration.

Our data on infectivity in *N. benthamiana *showed that ToLCPMV DNA A in association of DNA B of ToLCNDV induced typical disease symptoms. The same combination was tried in muskmelon to establish infectivity in this host. Inoculated as well as systemic leaves showed hypersensitive response (Figure 5A). In systemic leaves this appeared as leaves necrosis near the veins (Figure 5B). We have previously shown that the expression of NSP of ToLCNDV in transgenic plants induced hypersensitive response which was dependant on growth stage of plant [25]. We agroinfiltrated NSP of ToLCNDV cloned under 35S promoter while MP of ToLCNDV cloned under 35S promoter was used as control (Figure 6B). No effect on infiltration of 35S driven MP was found but the expression of NSP developed necrotic lesions that spread out of infiltration point (Figure 6B).

## Discussion

Phytopathogenic viruses are major constraints to agricultural productivity throughout the world. It is an unfortunate fact that Pakistan, in common with all other countries of southern Asia, is home to members of virtually all taxonomic groups of plant-infecting viruses [26-31] which, at least in part, explains the low agricultural productivity in the country. Prime amongst these viruses are the begomoviruses that have appeared as dominant pathogens on several crops. We show here that muskmelon samples previously found positive for ZYMV are co-infected with a begomovirus.

The complete nucleotide sequence of DNA A of begomoviruses is sufficient for identification and classification of begomoviruses. Viruses that share more than 90% sequence identity are considered as strain of the same virus while DNA sequence identity below 89% is considered as new species [32]. Based on this threshold value, the begomovirus identified in muskmelon is an isolate of ToLCPMV. The high levels of nucleotide sequence similarity, of both DNA A (86%) and DNAB (73%), between ToLCPMV and ToLCNDV indicate that they have a recent common ancestor and that ToLCPMV represents a lineage that has diverged from ToLCNDV. ToLCNDV has frequently been identified in cucurbits [33-38]. However, Koch's postulates for ToLCNDV causing diseases of cucurbits have not been satisfied. ToLCNDV is a cosmopolitan species that occurs throughout southern Asia, having been reported from Pakistan, India, Thailand and Taiwan [39-43]. However, since no efforts were made in these reports to inoculate clones on cucurbits, it is difficult to know whether an RNA virus was co-infecting these cucurbits.

The genome organization of ToLCPMV is the typical of Old World begomoviruses (having an AV2 gene) and phylogenetic analysis (Figure 3) show it and ToLCNDV to be most similar to (to group with) other begomoviruses with their center of diversity and origins lying in southern Asia [39,14-42,25]. Recombination contributes to the genetic diversification of geminivirus populations and has been related to the emergence of some serious plant diseases, as discussed earlier [44-48]. The prerequisite for recombination to occur is co-infection of the same cell of a host plant [49]. ToLCPMV DNA A has high levels of sequence identity to ToLCNDV-IN[PK:Kha:Chi:04] (accession number DQ116880) in the virion-sense (including the intergenic region) and to ToLCNDV-IN[IN:ND:AVT1] (AY428769) in the complementary-sense, indicating that this molecule is an intraspecific recombinant. The geminiviruses evolve/adapt by a number of mechanisms. Contrary to expectations, geminiviruses have nucleotide substitution rates that are comparable to those of RNA viruses [50]. Such high substitution rates would not be expected from a virus that utilizes host-encoded DNA polymerases with error correction. Additionally, recombination and component exchange (known as pseudo-recombination) are the major processes of geminivirus evolution [46,51-54]. A prime example of this is the ongoing pandemic of cassava mosaic disease that originated in northern Uganda, spread across eastern Africa and continues to spread throughout central and western Africa. The severe cassava mosaic disease of the pandemic is attributed to a recombinant strain of *East African cassava mosaic virus *known as the "Uganda Variant" [44].

Like the DNA A component, the DNA B component of ToLCPMV is a recombinant. The complementary-sense sequences (containing the MP gene) originate from ToLCNDV (showing the highest sequence identity with isolate ToLCNDV-IN (PK:Isl:T1/8:00; accession no. AY150304) while the intergenic region and virion-sense sequences are derived from an as yet undiscovered (or extinct) virus; having no high sequence similarity to any sequences in the databases. The DNA B of ToLCPMV is a mutant with a truncation of the NSP gene. It is unable to support symptoms development when inoculated to *N. benthamiana *in the presence of ToLCPMV DNA A. Mutational analysis suggests that the N-terminal sequences of NSP are involved in nuclear localization while C-terminal sequences are required for interaction with the MP [55]. Thus, truncation of ToLCPMV NSP at the N-terminus likely abolished the nuclear localization of the protein resulting in a defective molecule which cannot support infection or the protein is not produced at all since it is prematurely truncated. The NSP of ToLCNDV virus is a pathogenicity determinant where N-terminal sequences of protein are required for pathogenicity [25]. PCR amplification with universal and specific DNA B primers failed to show the presence of an intact DNA B in muskmelon samples, indicating that this defective molecule is the only DNA B present in muskmelon. We propose that truncated DNA B was selected on muskmelon probably because NSP was recognized by host defense system. Thus, DNA B with truncated NSP was maintained on muskmelon. A recent example of a begomovirus associated with resistance breakdown in cotton lacked an intact TrAP, probably to avoid host defense targeting the TrAP [56].

The integrity of the genomes of bipartite begomoviruses is maintained by them having compatible Rep binding sequences in the CR. Thus, the DNA A-encoded Rep is able to recognize and initiate the replication of both components [57-59,23]. Iteron sequences are usually species-specific, thus in most cases, the Rep of one virus would not be expected to recognize the iteron sequences of a distinct species [60-62]. The iteron sequences of ToLCNDV have been identified experimentally [63]. ToLCNDV and ToLCPMV have the same Rep binding sites and this likely explains our experimental finding that ToLCPMV DNA A is able to *trans*-replicate ToLCNDV DNA B and initiate a productive, symptomatic systemic infection of plants. ToLCNDV is a part of viral complex consisting of several DNA As that exchange DNA Bs readily. Here evidence is provided that, in addition to ToLCNDV and *Tomato leaf curl Gujarat virus *[64], the complex also encompasses ToLCPMV. This ability to readily form genomic reassortments (pseudo-recombination) is probably an adaptation that gives the virus complex an evolutionary advantage. Since DNA B components are involved in movement in plants [65,66], and thus host range determination [67-69], the ability to readily interact with distinct DNA B components will undoubtedly allow the viruses of the complex to alter/adapt their host ranges to take advantage of new niches.

For bipartite begomoviruses DNA B is considered as an integral part of the genome (a genomic component), rather than a satellite, due to the presence of the conserved region (CR) which is present in both components. Satellites are defined as molecules which share no significant sequence similarity with their helper viruses but require them for replication and movement in host plant [70]. Although clearly a DNA B, the lack of appreciable sequence similarity between the DNA A and DNA B components of ToLCPMV, with the exception of the iteron sequences and nonanucleotide sequence, means that the ToLCPMV DNA B component could be deemed a satellite. This contention is strengthened by the ability of the DNA A to readily exchange the component, a feature in common with satellites such as DNA betasatellite. Likely, in this case, the ToLCPMV DNA B is maintained as a satellite (although the question remains as to whether the NSP gene is expressed). Possibly then, in muskmelon, the ToLCPMV DNA B is maintained as a poorly-functional satellite which is not required for the infection. A recent analysis of DNA A and DNA B sequences has suggested that the two components differ in their evolution and DNA B may be considered as a satellite [71]. Further investigation will be needed to address this question. A possible explanation is that NSP being a pathogenicity determinant is the target of host defense as has been demonstrated previously [25]. Since all plants where ToLCPMV is found were earlier reported to be infected with ZYMV [10], there is a possibility that the defect in NSP is complemented by ZYMV. The presence of this defective DNA B in many plants and in geographically distant fields of muskmelon, as well as in various cucurbit species, however, suggests that this molecule may play an important part. A satellite that provides no selective advantage to a virus would be expected to be lost quite rapidly.

The data presented here show that ToLCPMV is widespread in the region that includes Iran, Pakistan and India and therefore an emerging virus on several crops. Unfortunately, the earlier reports have not demonstrated infectivity analysis and therefore, it is not possible to assess whether DNA B cloned from India and Iran was infectious. The role of ToLCPMV in muskmelon leaf curl disease remains to be demonstrated. We suggest that dual infection of a begomovirus with a defective DNA B and ZYMV may be important for disease symptoms. The lack of availability of infectious clones of ZYMV precludes us satisfying Koch's postulates for the disease in muskmelon. Our earlier results showed that mechanical inoculation of sap from infected muskmelon to healthy melon plants developed disease symptoms [10]. However, the possibility that begomovirus was also transmitted by sap inoculation cannot be ruled out. Our results show that ToLCPV is infectious to experimental hosts when inoculated with DNA B of ToLCNDV. Our recent results from cotton show that the begomovirus isolated from resistant cotton lack TrAP [56]. We hypothesized that begomoviruses may adopt to resistant host by truncation of an important gene. The maintenance of the defective DNA B in all field samples suggests a role for DNA B. It therefore appears that a synergistic interaction with ZYMV may have compensated for the defective NSP. We are trying to understand the possible synergistic interaction by cloning of helper component proteinase of ZYMV in a RNA virus vector such as PVX.

## Competing interests

The authors declare that they have no competing interests.

## Authors' contributions

AHM performed the experiments. AHM, RWB and SM were involved in data analysis experimental design and wrote the manuscript. All authors read and approved the final manuscript.

## References

[B1] Varma A Malathi VG Emerging geminivirus problems: a serious threat to crop production Ann Appl Biol 2003 142 145 164 10.1111/j.1744-7348.2003.tb00240.x

[B2] Fauquet CM Mayo MA Maniloff J Desselberge U Ball LA Virus Taxonomy: VIIIth Report of the International Committee on Taxonomy of Viruses" 2005 Elsevier Academic Press

[B3] Stanley J Bisaro DM Briddon RW Brown JK Fauquet CM Harrison BD Rybicki EP Stenger DC Fauquet CM, Mayo MA, Maniloff J, Desselberger U, Ball LA Geminiviridae Virus Taxonomy VIIIth Report of the ICTV 2005 London: Elsevier/Academic Press 301 326

[B4] Saunders K Bedford ID Briddon RW Markham PG Wong SM Stanley J A unique virus complex causes Ageratum yellow vein disease Proc Natl Acad Sci 2000 6890 6895 10.1073/pnas.97.12.6890 10841581 PMC18771

[B5] Mansoor S Briddon RW Zafar Y Stanley J Geminivirus disease complexes: an emerging threat Trends Plant Sci 2003 8 128 134 10.1016/S1360-1385(03)00007-4 12663223

[B6] Mansoor S Zafar Y Briddon RW Geminivirus disease complexes: the threat is spreading Trends in Plant Sci 2006 11 209 212 10.1016/j.tplants.2006.03.003 16616578

[B7] Mansoor S Briddon RW Fauquet CM Maintenance of an Old World betasatellite by a New World helper begomovirus and possible rapid adaptation of the betasatellite J Virol 2009 83 9347 9355 10.1128/JVI.00795-09 19570867 PMC2738271

[B8] Bull SE Briddon RW Markham PG Universal primers for the PCR-mediated amplification of DNA 1: a satellite-like molecule associated with begomovirus-DNA β complexes Mol Biotechnol 2003 23 83 86 10.1385/MB:23:1:83 12611272

[B9] Noueiry AO Lucas WJ Gilbertson RL Two proteins of a plant DNA virus coordinate nuclear and plasmodesmal transport Cell 1994 76 925 932 10.1016/0092-8674(94)90366-2 8124726

[B10] Malik AH Mansoor S Iram S Briddon RW Zafar Y A severe outbreak of melon yellow mosaic disease caused by zucchini yellow mosaic virus in Punjab province of Pakistan Plant Pathol 2006 55 285 10.1111/j.1365-3059.2006.01323.x

[B11] Doyle JJ Doyle JL Isolation of plant DNA from fresh tissue Focus 1990 12 13 15

[B12] Briddon RW Markham PG Universal primers for the PCR amplification of dicot-infecting geminiviruses Mol Biotechnol 1994 1 202 205 10.1007/BF02921559 7859161

[B13] Iram S Amrao L Mansoor S Malik AH Briddon RW Zafar Y First report of begomovrius associated with leaf curl disease of *Duranta erecta *in Pakistan Plant Pathol 2005 54 260 10.1111/j.1365-3059.2005.01129.x

[B14] Padidam M Beachy RN Fauquet CM Classification and identification of geminiviruses using sequence comparisons J Gen Virol 1995 76 249 263 10.1099/0022-1317-76-2-249 7844548

[B15] Thompson JD Gibson TJ Plewniak F Jeanmougin F Higgins DG The Clustal_X windows interface; flexible strategies for multiple sequence alignment aided by quality analysis tools Nucl Acid Res 1997 25 4876 4882 10.1093/nar/25.24.4876 PMC147148 9396791

[B16] Page RDM TREEVIEW: An application to display phylogenetic trees on personal computers Comput Appl Biosci 1996 12 357 358 8902363 10.1093/bioinformatics/12.4.357

[B17] Hellens RP Edwards EA Leyland NR Bean S Mullineaux PM pGreen: a versatile and flexible binary Ti Vector for Agrobacteruim-mediated plant transformation Plant Mol Biol 2000 42 819 832 10.1023/A:1006496308160 10890530

[B18] Hussain M Mansoor S Iram S Fatima AN Zafar Y The nuclear shuttle protein of Tomato leaf curl New Delhi virus is a pathogenicity determinant J Virol 2005 79 4434 4439 10.1128/JVI.79.7.4434-4439.2005 15767443 PMC1061533

[B19] Kumar Y Hallan V Zaidi AA Molecular characterization of a distinct bipartite begomovirus species infecting tomato in India Virus Genes 2008 37 425 431 10.1007/s11262-008-0286-1 18792773

[B20] Heydarnejad J Mozaffari A Massumi H Fazeli R Gray AJA Meredith S Lakay F Shepherd DN Martin DP Varsani A Complete sequences of tomato leaf curl Palampur virus isolates infecting cucurbits in Iran Arch Virol 2009 154 1015 1018 10.1007/s00705-009-0389-6 19424773

[B21] Laufs J Schumacher S Geisler N Jupin I Gronenborn B Identification of the nicking tyrosine of geminivirus Rep protein FEBS Lett 1995 377 258 262 10.1016/0014-5793(95)01355-5 8543063

[B22] Chatterji A Beachy RN Fauquet CM Expression of the oligomerization domain of the replication associated protein (Rep) of *Tomato leaf curl New Delhi virus *interferes with DNA accumulation of heterologous geminiviruses J Bio Chem 2001 276 25631 25638 10.1074/jbc.M100030200 11342533

[B23] Shivaprasad PV Thillaichidambaram P Balaji V Veluthambi K Expression of full-length and truncated Rep genes from *Mungbean yellow mosaic virus*-Vigna inhibits viral replication in transgenic tobacco Virus Genes 2006 33 365 374 10.1007/s11262-006-0077-5 16991009

[B24] Fontes EPB Luckow VA Hanley-Bowdoin L A geminivirus replication protein is a sequence-specific DNA binding protein Plant Cell 1994 4 597 608 10.1105/tpc.4.5.597 PMC160156 1498611

[B25] Hussain M Mansoor S Iram S Fatima AN Zafar Y The nuclear shuttle protein of Tomato leaf curl New Delhi virus is a pathogenicity determinant J Gen Virol 2005 79 4434 4439 10.1128/JVI.79.7.4434-4439.2005 PMC1061533 15767443

[B26] Mali VR Rajegore SB Occurrence of cucumber mosaic virus on banana in India Plant Dis 1979 63 138 142

[B27] Naidu RA Manohar SK Reddy DVR Reddy AS A plant rhabdovirus associated with peanut veinal chlorosis disease in India Plant Pathol 1989 38 623 626 10.1111/j.1365-3059.1989.tb01462.x

[B28] Ali A Natsuaki T Okuda S Identification and molecular characterization of viruses infecting cucurbits in Pakistan J Phytopathol 2004 152 677 682 10.1111/j.1439-0434.2004.00915.x

[B29] Raikhy G Hallan V Kulshrestha S Sharma ML Ram R Zaidi AA Molecular characterization of an Indian isolate of Carnation etched ring virus Acta Virol 2003 43 105 111 14524477

[B30] Varma R Prakash S Tomer SPS First report of Zucchini yellow mosaic virus in cucumber (*Cucumis sativus*) in India Plant Dis 2004 88 906 10.1094/PDIS.2004.88.8.906B 30812526

[B31] Mandal B Mandal S Pun KB Varma A First report of the association of a nanovirus with foorkey disease of large cardamom in India Plant Dis 2004 88 428 10.1094/PDIS.2004.88.4.428A 30812638

[B32] Fauquet CM Bisaro DM Briddon RW Brown JK Harrison BD Rybicki EP Stanley J Revision of taxonomic criteria for specie demarcation in the family *Geminiviridae *and an updated list of begomovirus species Arch Virol 2003 148 405 421 10.1007/s00705-002-0957-5 12557003

[B33] Maruthi MN Rekha AR Muniyappa V Pumpkin yellow vein mosaic disease is caused by two distinct begomoviruses: complete viral sequences and comparative transmission by an indigenous *Bemisia tabaci *and the introduced B-biotype EPPO Bull 2007 37 412 419 10.1111/j.1365-2338.2007.01127.x

[B34] Tahir M Haider MS First report of *Tomato leaf curl New Delhi virus *infecting bitter gourd in Pakistan Plant Pathol 2005 54 807 807 10.1111/j.1365-3059.2005.01215.x

[B35] Guzman P Sudarshana MR Seo YS Rojas MR Natwick E Turini T Mayberry K Gilbertson RL A new bipartite geminivirus (begomovirus) causing leaf curl and crumpling in cucurbits in the Imperial Valley of California Plant Dis 2000 84 488 10.1094/PDIS.2000.84.4.488C 30841181

[B36] Maruthi MN Colvin J Briddon RW Bull SE Muniyappa V Pumpkin yellow vein mosaic virus: a novel begomovirus infecting cucurbits J Plant Pathol 2003 85 64 65

[B37] Revill PA Ha CV Porchun SC Vu MT Dale JL The complete nucleotide sequence of two distinct geminiviruses infecting cucurbits in Vietnam Arch Virol 2003 148 1523 1541 10.1007/s00705-003-0109-6 12898329

[B38] Morales FJ Jones PG The ecology and epidemiology of whitefly-transmitted viruses in Latin America Virus Res 2004 100 57 65 10.1016/j.virusres.2003.12.014 15036836

[B39] Chatchawankanphanich O Chiang BT Green SK Singh SJ Maxwell DP Nucleotide sequence of a geminivirus associated with tomato leaf curl from India Plant Dis 1993 77 1168 10.1094/PD-77-1168C

[B40] Mansoor S Khan SH Saeed M Evidence for the association of a bipartite geminivirus with tomato leaf curl disease in Pakistan Plant Dis 1997 81 958 10.1094/PDIS.1997.81.8.958C 30866394

[B41] Mansoor S Khan SH Hussain M Zafar Y Pinner MS Briddon RW Stanley J Markham PG Association of a begomovirus and nanovirus like molecule with *Ageratum *yellow vein disease in Pakistan Plant Dis 2000 84 101 10.1094/PDIS.2000.84.1.101A 30841203

[B42] Samretwanich K Chiemsombat P Kittipakorn K Ikegami M Tomato leaf curl geminivirus associated with cucumber yellow leaf disease in Thailand J Phytopathol 2000 148 615 617

[B43] Haider MS Tahir M Latif S Briddon RW First report of *Tomato leaf curl New Delhi virus *infecting *Eclipta prostrata *in Pakistan Plant Pathol 2005 55 285 285 10.1111/j.1365-3059.2005.01278.x

[B44] Zhou X Liu Y Calvert L Munoz C Otim-Nape GW Evidence that DNA-A of a geminivirus associated with severe cassava mosaic disease in Uganda has arisen by interspecific recombination J Gen Virol 1997 78 2101 2111 9267014 10.1099/0022-1317-78-8-2101

[B45] Moffat AS Geminiviruses emerge as serious crop threat Science 1999 286 1835 10.1126/science.286.5446.1835

[B46] Paddidam M Sawyer S Fauquet CM Possible emergence of new geminiviruses by frequent recombination Virology 1999 265 218 225 10.1006/viro.1999.0056 10600594

[B47] Cui X Tao X Xie Y Fauquet CM Zhou X A DNA β associated with *Tomato yellow leaf curl China virus *is required for symptom induction J Virol 2004 78 13966 13974 10.1128/JVI.78.24.13966-13974.2004 15564504 PMC533896

[B48] Li ZH Xie Y Zhou XP Tobacco curly shoot virus DNA β is not necessary for infection but intensifies symptoms in a host-dependent manner Phytopathology 2005 95 902 908 10.1094/PHYTO-95-0902 18944412

[B49] Mansoor S Qazi J Amin I Khatri A Khan IA Raza S Zafar Y Briddon RW A PCR-based method, with internal control, for the detection of *Banana bunchy top virus *in banana Mol Biotehnol 2005 30 167 170 10.1385/MB:30:2:167 15920288

[B50] Duffy S Holmes EC Phylogenetic evidence for rapid rates of molecular evolution in the single-stranded DNA begomovirus *Tomato yellow leaf curl virus *(TYLCV) J Virol 2008 82 957 965 10.1128/JVI.01929-07 17977971 PMC2224568

[B51] Roye ME Mclaughlin WA Maxwell DP The evolution of new virus genes: Interspecies recombination among two geminiviruses from Jamaica Jamaican J Sci Technol 2000 11 42 46

[B52] Pita JS Fondong VN Sangre A Otim-Nape GW Ogwal S Fauquet CM Recombination, pseudorecombination and synergism of geminiviruses are determinant keys to the epidemic of severe cassava mosaic disease in Uganda J Gen Virol 2001 82 655 665 11172108 10.1099/0022-1317-82-3-655

[B53] Harrison BD Robinson DJ Another quarter century of great progress in understanding the biological properties of plant viruses Ann Appl Bio 2005 146 15 37 10.1111/j.1744-7348.2005.04111.x

[B54] Rothenstein D Haible D Dasgupta I Dutt N Patil BL Jeske H Biodiversity and recombination of cassava-infecting begomoviruses from southern India Arch Virol 2006 151 55 69 10.1007/s00705-005-0624-8 16132175

[B55] Sanderfoot AA Lazarowitz SG Getting it together in plant virus movement: Cooperative interactions between bipartite geminivirus movement proteins Trends Cell Biol 1996 6 353 358 10.1016/0962-8924(96)10031-3 15157433

[B56] Amrao L Akhter S Tahir MN Amin I Briddon RW Mansoor S Cotton leaf curl disease in Sindh province of Pakistan is associated with recombinant begomovirus components Virus Res 2010 153 161 165 10.1016/j.virusres.2010.07.003 20621137

[B57] Heyraud-Nitschke F Schumacher S Laufs J Schaefer S Schell J Gronenborn B Determination of the origin, cleavage and joining domains of geminivirus Rep proteins Nucleic Acids Res 1995 23 910 916 10.1093/nar/23.6.910 7731803 PMC306784

[B58] Laufs J Schumacher S Geisler N Jupin I Gronenborn B Identification of the nicking tyrosine of geminivirus Rep protein FEBS Lett 1995 377 258 262 10.1016/0014-5793(95)01355-5 8543063

[B59] Orozco BM Bowdoin HL A DNA structure is required for geminivirus origin function J Virol 1996 270 148 158 10.1128/jvi.70.1.148-158.1996 PMC189799 8523519

[B60] Choi IR Stenger DC Strain specific determinants of beet curly top geminivirus DNA replication Virology 1995 206 904 912 10.1006/viro.1995.1013 7856103

[B61] Jupin I Hericourt F Benz B Gronenborn G DNA replication specificity of TYLCV geminivirus is mediated by the amino-terminal 116 amino acids of the Rep protein FEBS Lett 1995 362 116 120 10.1016/0014-5793(95)00221-T 7720856

[B62] Gladfelter HJ Eagle PA Fontes EPB Batts LA Hanley-Bowdoin L Two domains of the AL1 protein mediate geminivirus origin recognition Virology 1997 239 186 197 10.1006/viro.1997.8869 9426458

[B63] Chatterji A Chaterji U Beachy RN Fauquet CM Sequence parameters that determine specificity of binding of the replication-associated protein to its cognate site in two strains of tomato leaf curl virus-New Delhi Virology 2000 273 341 350 10.1006/viro.2000.0434 10915605

[B64] Chakraborty S Pandey PK Banerjee MK Kallo G Fauquet CM Tomato leaf curl Gujarat virus, a new begomovirus species causing a severe leaf curl disease of tomato in Varanasi, India Virology 2003 93 1485 1495 10.1094/PHYTO.2003.93.12.1485 18943612

[B65] Sanderfoot AA Ingham DJ Lazarowitz SG A viral movement protein as a nuclear shuttle: The geminivirus BR1 movement protein contains domains essential for interaction with BL1 and nuclear localization Plant Physiol 1996 110 23 33 10.1104/pp.110.1.23 8587985 PMC157690

[B66] Rojas MR Hagen C Lucas WJ Gilbertson RL Exploiting chinks in the plant's armor: Evolution and emergence of geminiviruses Ann Rev Phytopathol 2005 43 361 394 10.1146/annurev.phyto.43.040204.135939 16078889

[B67] Berrie LC Rybicki EP Rey MEC Complete nucleotide sequence and host range of *South African cassava mosaic virus*: further evidence for recombination amongst begomoviruses J Gen Virol 2001 82 53 58 11125158 10.1099/0022-1317-82-1-53

[B68] Unseld S Ringel M Hofer P Hohnle M Jeske H Bedford ID Markham PG Frischmuth T Host range and symptom variation of pseudorecombinant virus produced by two distinct bipartite geminiviruses Arch Virol 2000 145 1449 1454 10.1007/s007050070101 10963348

[B69] Hofer P Engel M Jeske H Frischmuth T Nucleotide sequence of a new bipartite geminivirus isolated from the common weed *Sida rhombifolia *in Costa Rica J Gen Virol 1997 78 1785 1790 9225056 10.1099/0022-1317-78-7-1785

[B70] Briddon RW Stanley J Sub-viral agents associated with plant-infecting single-stranded DNA viruses Virology 2006 344 198 210 10.1016/j.virol.2005.09.042 16364750

[B71] Briddon RW Patil BL Bagewadi B Nawaz-ul-Rehman MS Fauquet CM Distinct evolutionary histories of the DNA-A and DNA-B components of bipartite begomoviruses BMC Evol Biol 2010 10 97 10.1186/1471-2148-10-97 20377896 PMC2858149

